# The Impact of Smoking on Arterial Stiffness in Young Adults: A Prospective Analysis

**DOI:** 10.3390/healthcare12191909

**Published:** 2024-09-24

**Authors:** Suzana Maria Guberna, Cosmina Elena Jercălău, Andreea Catană, Eleonora Drăgan, Anamaria-Georgiana Avram, Irina Cuciureanu, Maria Mirabela Manea, Cătălina Liliana Andrei

**Affiliations:** 1 Department of Cardiology, Emergency Hospital “Bagdasar-Arseni”, 050474 Bucharest, Romania; norisor13@yahoo.com (E.D.); anamaria.g.avram@gmail.com (A.-G.A.); irinadimitriu@yahoo.com (I.C.); catalina.andrei@umfcd.ro (C.L.A.); 2 Cardiology Department, Carol Davila University of Medicine and Pharmacy, 050474 Bucharest, Romania; catanaandreea91@yahoo.com (A.C.); maria.manea@umfcd.ro (M.M.M.); 3 Neurology Clinic, National Institute of Neurology and Neurovascular Diseases, 041915 Bucharest, Romania

**Keywords:** arterial stiffness, smoking, arteriograph, younger, personalized treatment

## Abstract

Background: Arterial stiffness is a crucial factor in the pathogenesis of cardiovascular disease, often associated with aging. However, the impact of smoking on arterial stiffness is frequently underestimated. This study aims to investigate the intricate relationship between smoking and arterial stiffness to advance our understanding of and therapeutic approaches to cardiovascular health. Methods: A prospective analysis was conducted from January to July 2024, focusing on arterial stiffness parameters in a cohort of students from the Carol Davila University of Medicine and Pharmacy. Participants were categorized as smokers or non-smokers based on self-reported smoking status. The study endpoints included correlations between high pulse wave velocity, elevated peripheral and central systolic blood pressure, increased peripheral and central pulse pressure, and smoking status. These markers were assessed using an arteriograph device measuring the time difference between the initial forward pulse wave and the reflected pulse wave in the brachial artery to indirectly estimate the PWV using oscillometric pulsations. Results: Our investigation, involving 102 young individuals aged 20 to 26 (69 females, 33 males), revealed that smokers exhibited significantly higher average values of arterial stiffness indicators compared to non-smokers. Current smokers had higher mean systolic blood pressure (130.65 vs. 123.05 mmHg), higher mean peripheral pulse pressure (53.19 vs. 45.64 mmHg), higher mean central pulse pressure (33.66 vs. 29.69 mmHg), and higher mean pulse wave velocity (5.27 vs. 5.03 m/s). Conclusions: The utilization of arterial stiffness markers as predictive tools offers opportunities for personalized treatment strategies, potentially enhancing cardiovascular health outcomes.

## 1. Introduction

Despite advancements in medical research and public health campaigns, smoking remains a prevalent and harmful habit that significantly impacts cardiovascular health [1,2]. One critical area of concern is the influence of smoking on arterial stiffness, a key factor in the development and progression of cardiovascular diseases [3,4]. Understanding the ongoing effects of smoking on arterial stiffness is vital for identifying high-risk individuals, implementing targeted interventions, and enhancing patient outcomes. Arterial stiffening, characterized by reduced artery elasticity, is commonly associated with aging and risk factors such as hypertension, diabetes mellitus, smoking, inflammation, and oxidative stress [5]. An increasing amount of research has recently revealed the enormous impact of smoking on arterial stiffness [6,7]. This loss of artery flexibility can impose additional stress on the heart, impair cardiac function, and contribute significantly to cardiovascular conditions like coronary artery disease (CAD) [8,9]. Previous research has highlighted the predictive significance of the PWV (pulse wave velocity) in cardiovascular disease risk. Studies have demonstrated that a higher baseline ePWV in the general population is linked to an increased risk of future cardiovascular events or mortality. This is attributable to the fact that heightened arterial stiffness can elevate systolic blood pressure, increase cardiac workload, potentially lead to left ventricular hypertrophy, and restrict coronary blood flow, all of which can contribute to the development of cardiovascular issues. Monitoring arterial stiffness regularly can provide valuable insights into the effectiveness of interventions, consequently enhancing patient care.

We have selected PWV because it is often considered the gold standard measurement for assessing arterial stiffness and is also sensitive to changes in arterial stiffness over time. This marker is not only an indicator of arterial stiffness but also a predictor of cardiovascular outcomes [10].

Integrating routine ePWV assessments into various clinical settings could enhance the cost-effectiveness of these measures and should be explored further in upcoming studies. While there is extensive research on the effects of smoking on the cardiovascular system, many studies have included confounding variables such as diabetes mellitus and alcohol consumption that can interfere with the impact of smoking on the cardiac system [4,11,12]. To address this gap, we conducted a study involving a sample of younger patients with no other comorbidities to minimize other influences on cardiac outcomes. By investigating the effects of smoking on arterial stiffness through this study, we aim to emphasize the importance of smoking cessation. Moving forward, finding effective ways to reduce arterial stiffness through lifestyle modifications, blood pressure management, and targeted medications may represent essential steps in promoting cardiovascular health and reducing the burden of cardiovascular diseases. Furthermore, beyond conventional approaches like lifestyle adjustments and medication, there is a compelling argument for delving into novel therapeutic avenues to combat arterial stiffness. Recent investigations into mitochondrial-targeted antioxidants have shown promise in reducing arterial stiffness [13]. By harnessing arterial stiffness markers as predictive tools, we open the door to personalized treatment strategies that could revolutionize cardiovascular health outcomes.

The objective of this study was to investigate the impact of smoking on parameters such as pulse wave velocity, peripheral and central systolic blood pressure, and peripheral and central pulse pressure. To mitigate confounding variables, we aimed to explore these associations in a cohort of young individuals.

## 2. Materials and Methods

Our study was prospective and conducted on a sample of students from the Carol Davila University of Medicine and Pharmacy during the period of January to July 2024, at the Cardiology Clinic of Bagdasar-Arseni Emergency Hospital in Bucharest. Participation was completely voluntary. The study included subjects aged between 18 and 30 years old at the time of registration. The inclusion criteria were as follows: (1) age between 18 and 30 years at the time of registration; (2) expression of agreement to participate in the study by signing the informed consent form; (3) absence of cardiovascular pathologies at the time of examination; and (4) absence of alcohol, coffee, and smoking before measurements, at least 30 min prior. Patients who did not meet these criteria were excluded from the study.

The initial evaluation included medical history, risk factors, lifestyle, heredocollateral antecedents (HCAs) of hypertension, myocardial infarction, stroke, diabetes, physical examination, and oral contraceptive treatments. Patient demographics and medical history were documented through a standardized questionnaire that inquired about any existing medical conditions, history of surgeries, and previous hospitalizations. Additionally, the weight, height, and abdominal circumference of each subject were recorded. Weight was measured using a medical scale, with the same device used for all subjects. Height measurements were taken with a graded centimeter, with subjects standing with heels together and back against a hard surface. To avoid measurement errors, two examiners performed these measurements for each subject. All measurements were assessed without shoes and with light clothing in the morning. Waist circumference was measured at the end of a normal expiration, horizontally at the top of the right iliac crest. Subsequently, blood pressure was evaluated using a sphygmomanometer and stethoscope, and all subjects underwent an arterial stiffness evaluation. Recordings were conducted in a calm environment, with the temperature being optimal to prevent arterial vasoconstriction (21–23 degrees Celsius). The subjects were briefed on the procedure and allowed to rest for 20 min before measurements were taken. Regarding the arteriograph, this device uses a cuff placed 2–3 cm above the elbow crease, following the path of the brachial artery. Data obtained through this device included the systolic blood pressure (SBP), diastolic blood pressure (DBP), left ventricular output (LVO), cardiac output (CO), cardiac index (CI), central systolic blood pressure (cSBP), central diastolic blood pressure (CDBP), pulse pressure (PP), central pulse pressure (CPP), pulse wave velocity (PWV), and augmentation index (AIx). The degree of arterial stiffness is determined by integrating all these determined parameters, with special weight placed on the values of the arterial velocity and augmentation index. These markers were assessed using an arteriograph device measuring the time difference between the initial forward pulse wave and the reflected pulse wave in the brachial artery to indirectly estimate the PWV using oscillometric pulsations. Using various mathematical formulas, the device converts the detected pulse wave velocities at the brachial level to approximate the pulse wave velocity at the central level, i.e., at the level of the aortic artery, providing a measure of the overall degree of arterial stiffness. Arterial stiffness can be additionally assessed by evaluating the pulse pressure (the difference between the systolic and diastolic blood pressure, where a value greater than 50 mmHg is considered pathological) and by calculating the augmentation index (the percentage increase in systolic pressure due to early reflection in the late systole of the reflected wave). Unlike other methods, which provide indirect measurements of arterial stiffness, arteriography offers a more comprehensive and detailed evaluation of the arterial structure and function. By directly imaging the arteries and identifying any abnormalities or changes in the arterial walls, arteriography can provide a more precise and accurate assessment of arterial stiffness.

This study was conducted according to the guidelines of the Declaration of Helsinki and approved by the Ethics Committee (protocol code 31014, date 10 July 2024).

Written informed consent was obtained from each subject.

### Statistical Methods

Continuous variables were expressed as mean ± standard deviation, and discrete variables were expressed as percentages. In this study, Student’s T-test, also known as the *t*-test, was used, and the Pearson correlation coefficient was also applied.

The graphical representations of the statistical results employed pie charts and scatter plots. In terms of the statistical significance of the regression model, a *p* value threshold of 0.05 was used in order to determine if the model was statistically significant.

## 3. Results

Our research involved 102 subjects. All patients were of Caucasian ethnicity. These individuals, falling within the age range of 21 to 26, presented a unique distribution: 6 individuals at 21 years old, 8 individuals at 22, 57 individuals at 23, 25 individuals at 24, 2 individuals at 25, and 4 individuals at 26 (Table 1).

Diving deeper into our participant demographics, our findings unveiled a gender composition of 69 females and 33 males, as demonstrated in Table 1.

Aligning with the World Health Organization’s guidelines on cardiovascular risk management, we emphasized the pivotal role of tailored moderate physical activity as a shield against cardiovascular events. This approach not only aids in the prevention of such incidents but also ameliorates cardiovascular conditions. Classifying our subjects based on their physical activity levels, we stratified them into three distinct categories: class I (less than 10 min of activity daily), class II (30 min daily), and class III (over 45 min daily). Varied physical activities—from leisurely strolls to intense workouts—were considered to cater to individual preferences, aligning with WHO recommendations [14].

Table 1 illuminates the final breakdown across the physical activity classes: 25.24% individuals in class I, 64.63% individuals in class II, and the remaining 13.13% individuals in class III. 

Furthermore, concerning smoking status, Table 1 also shows the following data: 31% of the participants stated that they are smokers, while the remaining 69% stated that they are non-smokers.

Given the fact that the smoking subjects are distributed within a very wide range in terms of the pack-year factor, it is necessary to add one more criterion, namely the division of smokers into those who have a consumption of less than 5 packs per year versus more than 5 packs per year. This represents another arbitrary parameter by which the duration and level of exposure to cigarette smoke felt by each individual subject can be evaluated.

As can be seen from Table 1, of the total number of smokers, the distribution of the group of smokers shows the following distribution: 34.37% have a consumption of more than 5 packs per year and 65.62% consume less than 5 packs per year.

A meticulous examination of Table 2 exposed noteworthy discrepancies in parameter values between non-smokers and smokers. Our statistical analyses unveiled significant variations in systolic blood pressure, central systolic blood pressure, pulse pressure, central pulse pressure, and pulse wave velocity, with a *p* value below 0.05. This underscores the stark impact of smoking on arterial stiffness. Additionally, alongside smoking, correlations with demographic factors like weight, waist circumference, and BMI were scrutinized, adding depth to our insights and emphasizing the multifaceted nature of arterial health.

The hemodynamic parameters in both smokers and non-smokers are outlined in detail in Table 3 and Table 4.

The results showed a significant difference in systolic blood pressure levels between smokers and non-smokers, with smokers exhibiting elevated levels. The statistical analysis yielded a low *p*-value of 0.0057, indicating a strong association between smoking and increased systolic blood pressure.

In contrast, there was no statistically significant difference in diastolic blood pressure levels between the two groups, suggesting that smoking may not have a significant influence on the diastolic blood pressure.

Similarly, there was no statistically significant difference in heart rate between smokers and non-smokers, indicating that smoking may not have a substantial impact on ventricular rates.

However, significant differences were observed in both the peripheral and central pulse pressure between the two groups. Smokers had higher pulse pressure levels compared to non-smokers, highlighting the significant impact of smoking on this cardiovascular parameter.

Furthermore, smokers exhibited higher pulse wave velocity values compared to non-smokers, indicating a significant effect of smoking on this cardiovascular parameter.

In contrast, there was no significant difference in the augmentation index between smokers and non-smokers, suggesting that smoking may not impact this parameter significantly.

Overall, these findings suggest that smoking has a significant influence on certain cardiovascular parameters, such as the systolic blood pressure, pulse pressure, and pulse wave velocity, while not significantly affecting others, such as the diastolic blood pressure, heart rate, and augmentation index.

### 3.1. The Correlation between Weight and Arterial Stiffness

#### The Correlation between Weight and PWV

**Figure 1:**
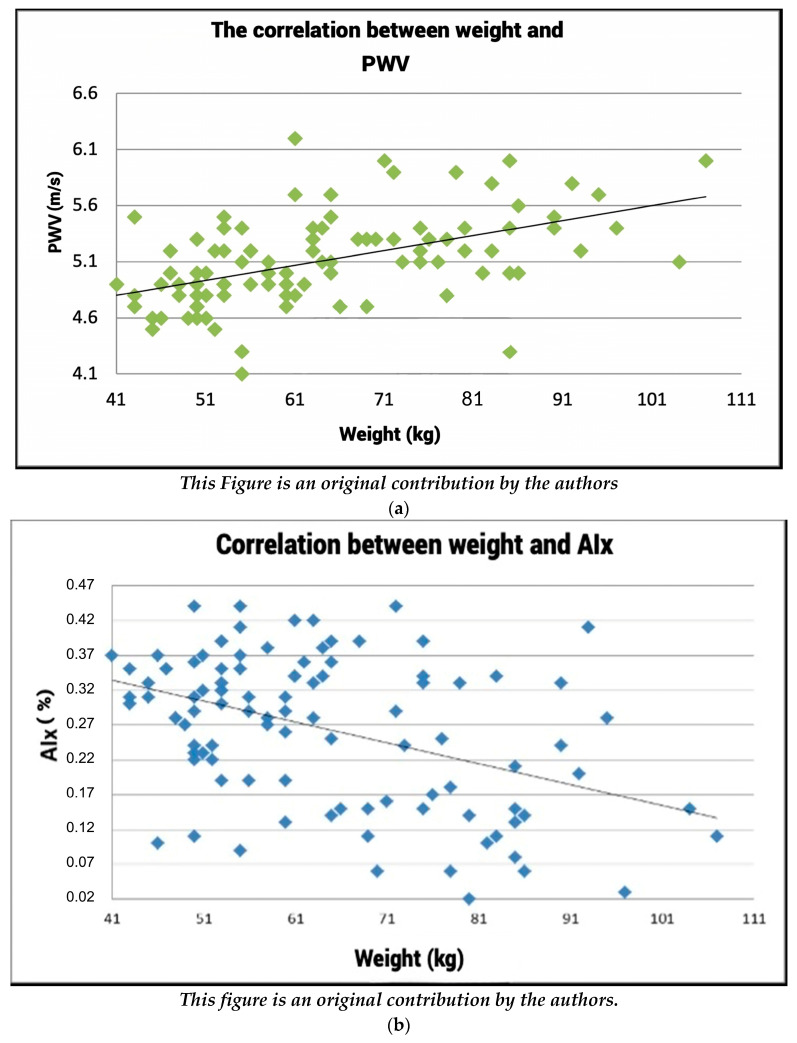
(**a**) Correlation between weight and PWV value. (**b**) Correlation between weight and AIx.

By meticulously computing the Pearson correlation index between the weight of the subjects enrolled in this study and the pulse wave velocity values measured in the same subjects, a correlation coefficient of r = −0.5081 was derived. This value suggests a notable positive linear correlation between the weight of the subjects and the corresponding pulse wave velocity values within the identical group of subjects (See Figure 1 for graphical representation).

Based on Figure 1a and the calculated Pearson correlation coefficient of r = −0.5081, a clear linear correlation emerges between the weight of subjects and their corresponding pulse wave velocity (PWV) values. Put simply, as the weight of individuals increases, there is a simultaneous rise in the recorded pulse wave velocity values, highlighting a positive correlation between these two variables.

Figure 1b unveils insights into the correlation between weight and the augmentation index (AIx). The Pearson correlation index value of r = −0.4192 signifies a negative linear correlation between weight and the AIx. Essentially, as the weight of subjects varies, there is a concomitant alteration in the AIx values in an inverse direction, elucidating a negative correlation trend between weight and the AIx.

### 3.2. The Correlation between Body Mass Index and Arterial Stiffness

#### 3.2.1. The Correlation between Body Mass Index and PWV

**Figure 2:**
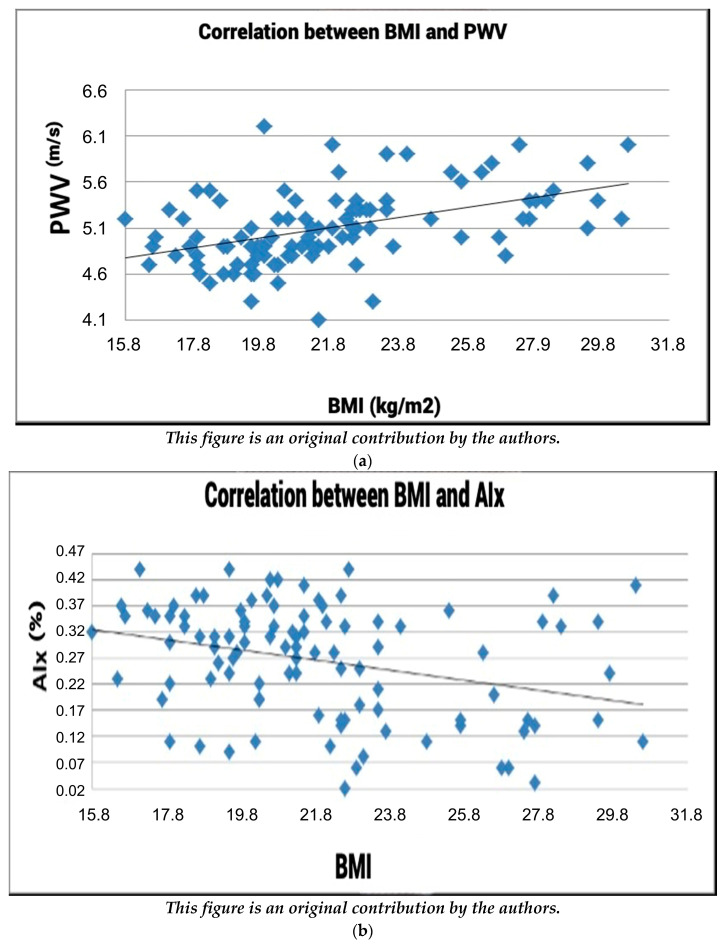
(**a**) Correlation between BMI and PWV. (**b**) Correlation between BMI and AIx.

Analyzing the relationship between body mass index (BMI) and pulse wave velocity (PWV), as depicted in Figure 2a, the Pearson correlation index of r = 0.4829 denotes a positive linear correlation between these variables. This implies that there is a direct relationship between the body mass index values of subjects and their corresponding pulse wave velocity readings. This correlation signifies that as the body mass index of subjects increases, there is a corresponding elevation in pulse wave velocity readings. In essence, higher BMI values are associated with heightened pulse wave velocity values, showcasing a positive relationship between these two variables.

#### 3.2.2. Evaluating Body Mass Index vs. Augmentation Index (AIx)

Figure 2b delineates the correlation between body mass index and the augmentation index (AIx). With a Pearson correlation index value of r = −0.2972, a negative correlation between the body mass index and AIx is indicated. This negative correlation suggests that fluctuations in the body mass index value lead to changes in AIx values in an inverse manner, highlighting a negative linear relationship between body mass index and AIx.

### 3.3. The Correlation between Pack-Years and Arterial Stiffness

#### 3.3.1. Exploring the Pack-Years vs. Pulse Wave Velocity Correlation (Figure 3a)

**Figure 3:**
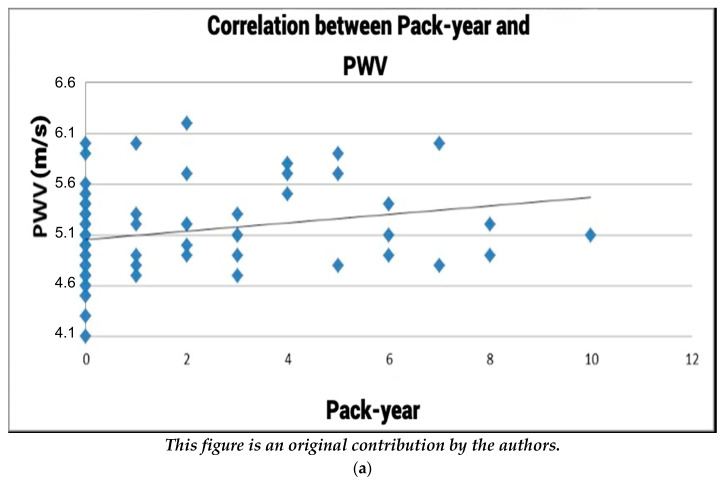

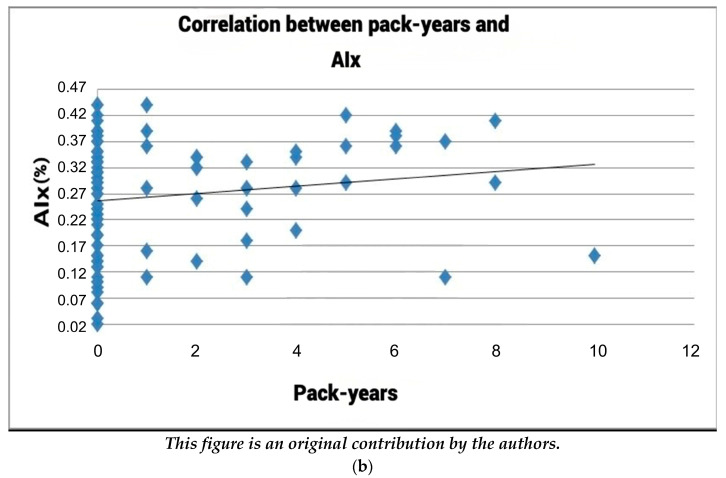
(**a**) Correlation between pack-years and PWV. (**b**) Correlation between pack-years and AIx.

The concept of smoking intensity, as quantified by pack-years, serves as a pivotal factor in analyzing the correlation between smoking and arterial stiffness. In the context of the correlation between pack-years and pulse wave velocity (PWV), depicted in Figure 3, the Pearson correlation index of r = 0.2497 indicates a positive correlation between pack-years and PWV. This positive correlation implies that as the pack-year value, representing smoking intensity, increases, there is a concomitant rise in pulse wave velocity readings. Essentially, higher pack-year values are associated with elevated pulse wave velocity, underlining a positive correlation between smoking intensity and arterial stiffness. In essence, as individuals accumulate more pack-years or years of smoking, there is a corresponding linear escalation in pulse wave velocity, underscoring the impact of smoking exposure on arterial stiffness and cardiovascular health.

#### 3.3.2. The Correlation between Pack-Years and the AIx

Examining the correlation between pack-years and the augmentation index (AIx), represented in Figure 3b, the Pearson correlation index value of r = 0.2042 indicates a positive correlation between smoking exposure, quantified by pack-years, and the augmentation index. This positive correlation suggests that as individuals accrue more pack-years, reflecting greater exposure to smoking, there is a corresponding increase in the augmentation index. Essentially, higher levels of smoking exposure, measured through pack-years, are associated with elevated augmentation index values, emphasizing a positive correlation between smoking intensity and arterial stiffness, as indicated by the AIx.

With both the pulse wave velocity and the augmentation index displaying positive linear correlations with pack-years, it can be deduced that exposure to cigarette smoke, quantified by pack-years and years of smoking, correlates positively with arterial stiffness. Essentially, heightened exposure to cigarette smoke is associated with a more pronounced level of arterial stiffness, reflecting the impact of smoking on cardiovascular health.

## 4. Discussion

Our investigation, conducted on a cohort of young individuals aged 20 to 26, unveiled that smoking triggers significant alterations in various cardiovascular parameters (such as the pulse wave velocity, peripheral systolic blood pressure, central systolic blood pressure, peripheral pulse pressure, and central pulse pressure). In our study, current smokers had higher mean systolic blood pressure (130.65 vs. 123.05 mmHg), higher mean peripheral pulse pressure (53.19 vs. 45.64 mmHg), higher mean central pulse pressure (33.66 vs. 29.69 mmHg), and higher mean pulse wave velocity (5.27 vs. 5.03 m/s). These changes heighten the susceptibility to arterial stiffness, thereby increasing the risk of developing a range of cardiovascular pathologies including myocardial infarction, hypertension, and stroke. While central pressures are typically directly measured using invasive devices, several techniques have emerged to indirectly estimate central pressures by analyzing applanated carotid and radial pulses or carotid distension waves, with the arteriograph being one such device [15]. The pulse wave velocity (PWV), a critical marker of arterial stiffness, measures the rate at which pressure waves propagate through the arteries. The arteriograph technology utilizes oscillometric pulsations in the brachial artery to indirectly estimate the PWV by measuring the time lag between the initial (forward) and reflected pulse waves. Unlike methods that involve ECG recording, the arteriograph does not require this. With the convenience of using a single cuff for data collection, the arteriograph offers higher practicality, although sophisticated signal processing is necessary to extract the required parameters [16]. The augmentation index signifies the discrepancy between the first and second peak of systolic blood pressure in relation to the pulse pressure. Its measurement is predicated on identifying the reflected wave that returns to the heart during each cardiac systole. In stiff arteries, the reflected wave returns to the left ventricle faster, causing an amplification of blood pressure. This accelerated return necessitates more oxygen at the cardiac level, amplifying the cardiac workload to counter the afterload, a consequence of arterial stiffness. Consequently, the augmentation index serves as a valuable assessment tool for arterial stiffness, alongside the pulse wave velocity [17].

Smoking, a major concern in global public health initiatives, has undergone extensive scrutiny for its adverse effects on vascular endothelial function. Studies have demonstrated that cigarette smoking disrupts the endothelial equilibrium by diminishing nitric oxide (NO) availability in blood vessels, primarily induced by oxidative stress and inflammation.

NO plays a crucial role in maintaining various endothelial functions such as vasodilation, anti-thrombotic responses, anti-inflammatory effects, and antioxidant defense mechanisms. However, endothelial dysfunction resulting from diminished nitric oxide levels leads to the development of atherosclerotic plaques, causing structural changes in arterial walls and an increase in arterial stiffness [18,19].

Notably, arterial stiffness is recognized as an independent risk factor for cardiovascular disease (CVD) and overall mortality, even after adjusting for traditional cardiovascular risk factors [20].

An assessment of arterial stiffness can be conducted using measures like the pulse wave velocity or the augmentation index, which reflects the wave reflection and serves as an indirect marker of arterial stiffening [21]. Our research illuminates the influence of smoking on cardiovascular health by confirming a direct positive relationship between smoking and the pulse wave velocity, peripheral and central systolic blood pressure, as well as peripheral and central pulse pressure. Additionally, our research reveals that demographic variables such as weight, body mass index, and abdominal circumference exhibit a positive linear relationship with the pulse wave velocity and a negative linear relationship with the augmentation index. The pack-year factor, quantifying exposure to cigarette smoke, also shows a positive linear correlation with both the pulse wave velocity and augmentation index. In recent years, numerous studies have explored the effects of smoking on blood pressure, yielding varied outcomes. While some epidemiological investigations suggest that smokers have lower blood pressure levels than non-smokers, others indicate an elevated risk of developing hypertension among smokers [22,23,24,25].

Our findings align with the latter research, demonstrating a positive association between tobacco use and peripheral and central systolic blood pressure.

Previous studies have predominantly examined the impact of smoking on blood pressure levels, often overlooking potential confounding variables that could influence the relationship between smoking and blood pressure [4,11,12]. In the realm of tobacco use and cardiovascular outcomes, it becomes paramount to account for diverse confounding factors that may shape this intricate association. In our study, the participants were predominantly younger and in good health, characterized by a functional baroreflex axis. This contrasts with individuals affected by conditions like hypertension, diabetes mellitus, and heart failure, who typically manifest baroreflex dysfunction and heightened vulnerability to the effects of smoking. This age-related divergence may partly elucidate the variances observed in muscle sympathetic nerve activity (MSNA) and skin sympathetic nerve activity (SSNA) responses to acute smoking exposure between our study and previous research [26].

Our investigation highlighted a distinct positive correlation between the pulse pressure and blood pressure, aligning seamlessly with findings from the existing literature. Furthermore, our research reinforced the robust relationship between smoking and PWV, which is consistent with prior studies that underscore the prognostic value of elevated PWV. Studies have established that a higher baseline pulse wave velocity is associated with an escalated risk of future cardiovascular events or mortality, underscoring the significance of arterial stiffness as a predictive marker for adverse outcomes [10,27].

### 4.1. Limitations

In our research, we encountered several limitations. Initially, this study was confined to a single center with a relatively limited patient cohort, emphasizing the need for future extensive, cross-center studies. Furthermore, arterial stiffness can vary due to factors such as circadian rhythms, diet, stress, and other temporary influences, which can impact the accuracy and reliability of measurements. Our research primarily focuses on healthy subjects and examines both peripheral and central pulse pressure in the absence of pathological conditions. Given the recent clinical findings, additional research is necessary to establish the correlation between pulse pressure amplification and pathological conditions in cardiology. Expanding our research to include individuals with pathological cardiovascular conditions will enable us to develop a more robust understanding.

Another constraint of the study was that the majority of individuals reported consuming only 10 packs of cigarettes per year. However, it is important to note that there are individuals who consume even larger quantities in reality. Therefore, future research should consider including participants who smoke many packs per year to provide a more comprehensive understanding of the topic. Additionally, other limitations that can be noted include a predominantly female population and unknown smoking duration among individuals.

Furthermore, with the convenience of using a single cuff for data collection, the arteriograph offers higher practicality, but sophisticated signal processing is necessary to extract the required parameters.

### 4.2. Future Directions

While advocating for smoking cessation continues to be a critical component in enhancing cardiovascular health, it is imperative to consider exploring alternative strategies to mitigate arterial stiffness. Finding innovative solutions to address arterial stiffness could potentially provide new avenues for intervention. Researching and developing treatments that are tailored to reducing arterial stiffness in individuals who face challenges in quitting smoking or who remain at risk despite efforts to abstain from tobacco use may offer supplementary support in the future.

## 5. Conclusions

In conclusion, this study represents a significant advancement in cardiovascular care, with the potential to reshape current patient treatment protocols. The incorporation of pulse wave velocity assessments into standard evaluations not only shows promise in enhancing the management of high-risk patients but also paves the way for innovative research into new medications aimed at reducing arterial stiffness. Furthermore, the observed correlation between an improved augmentation index and PWV with decreased arterial stiffness highlights the critical importance of addressing arterial health in patient care. By prioritizing the enhancement of these parameters, it is conceivable to lower arterial stiffness and ultimately improve the overall patient outcomes. The potential for the development of novel therapeutic approaches inspired by these findings signifies a promising advancement in the field of cardiology. As we delve further into the implications of this research, emphasizing arterial health through advanced assessments like PWV evaluations has the potential to lead to a more effective and personalized approach to cardiovascular care. This shift towards a more comprehensive understanding and management of arterial health could significantly impact patient outcomes and pave the way for future advancements in cardiovascular medicine.

## Figures and Tables

**Table 1:** Clinical and demographical characteristics of the participants.

Characteristic	Frequency
Gender	
Female	69
Male	33
Age	
21	6%
22	8%
23	56%
24	24%
25	2%
26	4%
Physical activity level	
I	25.24%
II	64.63%
III	13.13%
Smoking status	
Smokers	31%
Non-smokers	69%
Smokers	
More than 5 packs/year	34.37%
Less than 5 packs/year	65.62%

**Table 2:** Representation of the mean values of the detected parameters.

Parameter	Smoker	Non-Smoker	*p* Value
Systolic BP	130.65 ± 14.3 mmhg	123.05 ± 11.72 mmhg	0.0057
Diastolic BP	77.43 ± 7.46 mmhg	77.34 ± 7.88 mmhg	0.8583
Heart rate	89.81 ± 12.64 b/min	90.15 ± 14.1 b/min	0.9854
Central systolic BP	113.13 ± 10.51 mmhg	108.93 ± 8.8 mmhg	0.0400
Central diastolic BP	79.47 ± 7.48 mmhg	79.24 ± 7.98 mmhg	0.7990
Pulse pressure	53.19 ± 14.99 mmhg	45.64 ± 10.39 mmhg	0.0054
Central pulse pressure	33.66 ± 8.06 mmhg	29.69 ± 5.94 mmhg	0.0103
Cardiac output	5.18 ± 0.84 L/min	5.04 ± 0.7 L/min	0.4191
Pulse wave velocity	5.27 ± 0.42 m/s	5.03 ± 0.35 m/s	0.0035
Augmentation index	0.29 ± 0.09%	0.25 ± 0.1%	0.0888

**Table 3:** Hemodynamic parameters in smokers.

	Minimum Value	Medium Value	Maximum Value	Standard Deviation
Systolic blood pressure	111	130.65	167	±14.3 mmHg
Diastolic blood pressure	55	77.43	95	±7.46 mmHg
Heart rate	71	89.81	117	±12.64 mmHg
Central systolic blood pressure	98	113.13	143	±10.51 mmHg
Central diastolic blood pressure	59	79.47	98	±7.48 mmHg
Periphereal pulse pressure	31	53.19	92	±14.99 mmHg
Central pulse pressure	21	33.66	56	±8.06 mmHg
Cardiac output	3.4 L/min	5.18 L/min	7.7 L/min	±0.84 L/min
Pulse wave velocity	4.7 m/s	5.27 m/s	6.2 m/s	±0.42 m/s
Augmentation index	0.11%	0.29%	0.44%	±0.09%

**Table 4:** Hemodynamic parameters in non-smokers.

	Minimum Value	Medium Value	Maximum Value	Standard Deviation
Systolic blood pressure	91	123.05	160	±11.72 mmHg
Diastolic blood pressure	55	77.34	95	±7.88 mmHg
Heart rate	66	90.15	122	±14.1 mmHg
Central systolic blood pressure	87	108.93	130	±8.8 mmHg
Central diastolic blood pressure	55	79.24	96	±7.98 mmHg
Periphereal pulse pressure	27	45.64	76	±10.39 mmHg
Central pulse pressure	18	29.69	50	±5.94 mmHg
Cardiac output	3.7 L/min	5.04 L/min	7.2 L/min	±0.7 L/min
Pulse wave velocity	4.1 m/s	5.03 m/s	6 m/s	±0.35 m/s
Augmentation index	0.02%	0.25%	0.44%	±0.1%

## Data Availability

The data presented in this study are available on request from the corresponding author. The data are not publicly available due to privacy issues.

## Abbreviations

Augmentation index	AIx
Body mass index	BMI
Pulse wave velocity	PWV
Nitric oxide	NO
Cardiovascular disease	CVD
Muscle sympathetic nerve activity	MSNA
Skin sympathetic nerve activity	SSNA
Systolic blood pressure	SBP
Diastolic blood pressure	DBP
Left ventricular output	LVO
Cardiac output	CO
Cardiac index	CI
Central systolic blood pressure	cSBP
Central diastolic blood pressure	CDBP
Pulse pressure	PP
Central pulse pressure	CPP
